# COVID-19 Vaccination Acceptance and Hesitancy in Dialysis Staff: First Results From New York City

**DOI:** 10.1016/j.ekir.2021.02.001

**Published:** 2021-02-12

**Authors:** Gemma M. Pamplona, Terry Sullivan, Peter Kotanko

**Affiliations:** 1 Renal Research Institute, New York, New York, USA; 2 Division of Nephrology, Icahn School of Medicine at Mount Sinai, New York, New York, USA

To the Editor:

Broad adoption of vaccination against severe acute respiratory syndrome coronavirus 2 (SARS-CoV-2) is key to successfully fighting the spread of coronavirus disease 2019 (COVID-19). When fallen ill with COVID-19, in-center hemodialysis patients are at particularly high risk for morbidity and mortality.^1^^,^^2^ Therefore, attaining high vaccination rates in both dialysis patients and staff is of utmost importance.

The New York State Department of Health COVID-19 vaccination plan calls for a phased distribution of the vaccine (https://covid19vaccine.health.ny.gov/phased-distribution-vaccine). In accordance with that plan, all outpatient/ambulatory front-line, high-risk health care workers of any age who provide direct in-person patient care are eligible for vaccination; this definition includes dialysis staff.

Here we report dialysis staff vaccination acceptance and hesitancy rates from 4 Renal Research Institute dialysis clinics and a home dialysis program located in New York, New York. Inoculation of the first dose was done between 13 and 21 January 2021. The staff count was 157, including full-time and part-time employees, temporary workers, and *per diem* staff. Staff who were pregnant or breast feeding, were on leave of absence, had contracted COVID-19 less than 90 days ago, or explicitly expressed vaccination hesitancy were not offered inoculation. Staff with a history of confirmed COVID-19 more than 90 days ago or at some unknown time in the past were offered vaccination; while in principle willing to get inoculated, these staff members wished to receive the vaccine later. Six employees (3.8%) explicitly expressed vaccination hesitancy (Table 1).

**Table 1:** Vaccination of Renal Research Institute dialysis staff in New York City, New York, January 2021

Staff	Not vaccinated (n = 42 [26.8])						Vaccinated^b^
	Leave of absence	Pregnancy or breast feeding	Past COVID-19 infection			Vaccination hesitancy	
			>90 days	≤90 days	Unknown^a^		
157	4 (2.5)	8 (5.1)	14 (8.9)	6 (3.8)	4 (2.5)	6 (3.8)	115 (73.2)

COVID-19, coronavirus disease 2019; SARS-CoV-2, severe acute respiratory syndrome coronavirus 2.

Data are presented as number (%).

a These individuals had tested positive for SARS-CoV-2 antibodies but had no confirmed COVID-19 infection.

b This number includes 3 staff members who were vaccinated by a different health care provider.

In preparation of the vaccination rollout, we have undertaken several measures to attain a low vaccination hesitancy rate, including extensive information on vaccination and in-person briefings, if requested. The vaccination was endorsed unanimously by medical and staff leadership. Also, the fact that New York City was hit hard by the pandemic and that many staff members have witnessed COVID-19 firsthand in patients, family, and friends may have contributed to the high vaccination rate. Finally, since spring 2020, clinic staff have been actively involved in quality improvement and clinical research projects related to COVID-19.^3^^,^^4^ We believe that these factors in aggregate contributed to the high vaccination acceptance rate we observed.

Lastly, there are additional opportunities to increase vaccination rates. First, because reinfection with COVID-19 is possible, the Centers for Disease Control and Prevention recommends that the vaccine should be offered to individuals with previous COVID-19 infection. However, while vaccine supply remains limited, persons with recent documented acute SARS-CoV-2 infection may choose to temporarily delay vaccination (https://www.cdc.gov/coronavirus/2019-ncov/vaccines/facts.html). Second, it will be important to communicate that pregnant women may choose to be vaccinated.

## Disclosure

The authors are employees of the Renal Research Institute, a wholly owned subsidiary of Fresenius Medical Care (FMC). PK and TS hold stock in FMC.
