# Supplementary material for: COVID-19 Vaccination Acceptance and Hesitancy in Dialysis Staff: First Results From New York City
Source: Kidney Int Rep. 2021 Feb 12;6(4):1192–3. doi: 10.1016/j.ekir.2021.02.001 (PMC7879030; doi:10.1016/j.ekir.2021.02.001)
Supplement: Supplementary File (PDF) [file mmc1.pdf]

Supplementary Material

Methods

We report dialysis staff vaccination acceptance and hesitancy rates from four Renal Research Institute dialysis clinics and a home dialysis program located in New York City, NY. Inoculation of the 1st dose was done between January 13th and 21st, 2021. The staff count was 157, including full time and part-time employees, temps, and per diem staff. Staff who were pregnant or breast feeding, were on leave of absence, suffered from COVID-19 less than 90 days ago or expressed vaccination hesitancy explicitly were not offered inoculation. Staff with a history of confirmed COVID-19 more than 90 days ago or at some unknown time in the past were offered vaccination.
